# 6-shogaol against 3-Nitropropionic acid-induced Huntington’s disease in rodents: Based on molecular docking/targeting pro-inflammatory cytokines/NF-κB-BDNF-Nrf2 pathway

**DOI:** 10.1371/journal.pone.0305358

**Published:** 2024-07-15

**Authors:** Ebtihaj J. Jambi, Abdulaziz Alamri, Muhammad Afzal, Fahad A. Al-Abbasi, Salwa D. Al-Qahtani, Naif A. R. Almalki, Azizah Salim Bawadood, Sami I. Alzarea, Nadeem Sayyed, Imran Kazmi

**Affiliations:** 1 Department of Biochemistry, Faculty of Sciences, King Abdulaziz University, Jeddah, Saudi Arabia; 2 Experimental Biochemistry Unit, King Fahd Medical Research Center, King Abdulaziz University, Jeddah, Saudi Arabia; 3 Department of Biochemistry, College of Science, King Saud University, Riyadh, Saudi Arabia; 4 Department of Pharmaceutical Sciences, Pharmacy Program, Batterjee Medical College, Jeddah, Saudi Arabia; 5 Department of Medical Laboratory Sciences, College of Applied Medical Sciences, Majmaah University, Al Majmaah, Saudi Arabia; 6 Basic Medical Sciences Department, College of Medicine, Prince Sattam Bin Abdulaziz University, Al-Kharj, Saudi Arabia; 7 Department of Pharmacology, College of Pharmacy, Jouf University, Aljouf, Sakaka, Saudi Arabia; 8 School of Pharmacy, Glocal University, Saharanpur, India; King Abdulaziz University Faculty of Medicine, SAUDI ARABIA

## Abstract

**Background:**

Huntington’s disease (HD) is an extremely harmful autosomal inherited neurodegenerative disease. Motor dysfunction, mental disorder, and cognitive deficits are the characteristic features of this disease. The current study examined whether 6-shogaol has a protective effect against 3-Nitropropionic Acid (3-NPA)-induced HD in rats.

**Methods:**

A total of thirty male Wistar rats received 6-shogaol (10 and 20 mg/kg, per oral) an hour before injection of 3-NPA (10 mg/kg i.p.) for 15 days. Behavioral tests were performed, including narrow beam walk, rotarod test, and grip strength test. Biochemical tests promoting oxidative stress were evaluated [superoxide dismutase (SOD), reduced glutathione (GSH), catalase (CAT) and malondialdehyde (MDA)], including changes to neurotransmitters serotonin (5-HT), dopamine (DA), norepinephrine (NE), homovanillic acid (HVA), (3,4-dihydroxyphenylacetic acid (DOPAC), γ-aminobutyric acid (GABA), and 5-hydroxy indole acetic acid (5-HIAA), nuclear factor kappa-B (NF-κB), tumor necrosis factor-α (TNF-α), interleukins-1β (IL-1β), IL-6, brain-derived neurotrophic factor (BDNF), and nuclear factor erythroid 2-related factor 2 (Nrf2). The 6-shogaol was docked to the active site of TNF-α (2AZ5), NF-κB (1SVC), BDNF) [1B8M], and Nrf2 [5FZN] proteins using AutoDock tools.

**Results:**

The 6-shogaol group significantly improved behavioral activity over the 3-NPA-injected control rats. Moreover, 3-NPA-induced significantly altered neurotransmitters, biochemical and neuroinflammatory indices, which could efficiently be reversed by 6-shogaol. The 6-shogaol showed favorable negative binding energies at -9.271 (BDNF) kcal/mol.

**Conclusions:**

The present investigation demonstrated the neuroprotective effects of 6-shogaol in an experimental animal paradigm against 3-NPA-induced HD in rats. The suggested mechanism is supported by immunohistochemical analysis and western blots, although more research is necessary for definite confirmation.

## Introduction

Huntington’s disease (HD) is an extremely harmful autosomal inherited neurodegenerative disease. Motor dysfunction, mental disorders, and cognitive deficits are the characteristic features of this disease. Although the striatum is crucial for motor regulation and learning activities, neuronal damage, particularly in the striatum, is the main feature of HD [1]. The person in the mature phase had significant rigidity difficulties, minimal mobility, synchronization issues, sporadic convulsions, ambiguous articulation, feeding difficulties, impaired judgment, clouded thinking, and initial psychological behavior problems [2, 3]. HD is a neurological disorder associated with memory loss and sensory decline, which ultimately results in death [4]. The most typical symptoms of HD include deficits in cognitive, motor, and psychosocial functions. People with HD begin to show symptoms at a young age, and the condition eventually results in mortality due to its progressive development that takes place over many years [5].

Previous research has revealed several neurotransmitter changes in HD caused by mutant huntingtin protein (mHTT), including changes in γ-aminobutyric acid (GABA) and glutamate. Furthermore, it was discovered that mHTT in HD patients is connected with significant modifications in Bcl-2-associated X-protein (Bax), brain-derived neurotrophic factor (BDNF), Bid, intracellular calcium levels, and N-methyl-D-aspartate (NMDA) receptors [6, 7]. Moreover, research on rodents revealed a dramatic energy imbalance between supply and demand that led to mitochondrial malfunction in HD and altered normal neural functions [8, 9].

Succinate dehydrogenase, the electron transport chain enzyme, and an enzyme of the tricyclic antidepressant cycle are all permanently inhibited by the mitochondrial neurotoxic 3-nitropionic acid (3-NP) [10, 11]. Hydroxyl-free radicals and superoxide, which are produced in the body, can harm deoxyribonucleic acid (DNA), proteins, lipids, affecting brain structure, neurotransmitter levels, and impact on inflammation [12]. Reactive oxygen species (ROS) have been identified as potential regulators of neuronal cell death in HD [13]. In the striatum, systemic treatment of 3-NP causes central nervous system (CNS) injuries that specifically target medium spiny neurons, demonstrating the spatial and neuronal selectivity of HD [14].

Several studies have linked the neurotoxicity of 3-NP to an increase in the production of superoxide (O2-) and hydroxyl free radicals (OH-) in the striatum, as well as markers of oxidative damage in the CNS [15]. In addition to raising or activating tumor necrosis factor (TNF-α), Interleukin-1 beta (IL-1β), NMDA, and oxidative stress promote neuroinflammation [16]. DNA fragmentation was more prevalent, and the production of apurinic or apyrimidinic endonucleases was lower [17, 18], showing that rats over the age are more susceptible to the oxidative damage produced by 3-NP. Reduced levels of striatal catalase (CAT), superoxide dismutase (SOD), and glutathione (GSH), as well as 3-NP, suggest a deficiency in antioxidant defenses [19].

The herb *Zingiber officinale*, a perennial (Zingiberaceae family), and its broad tuberous rhizomes are broadly used for medicinal purposes [20]. Ginger is reported to include a variety of distinct substances, with paradols, gingerols, diarylheptanoids, and shogaols [21]. Consequently, further research is required to determine ginger’s probable active ingredient that has the ability to treat Alzheimer’s disease and improve memory. It is well-known that ginger contains several important active ingredients, including 6-shogaol [22–24]. It has been demonstrated that 6-shogaol, in particular, prevents endothelial cells from harm caused by Aβ [25]. 6-Shogaol is a single molecule with a specific structure composed of carbon, hydrogen, and oxygen atoms. It is a chemical compound with the formula C17H24O3 and belongs to a class of compounds known as phenolic alkanones, which are characterized by their strong flavor and aroma. 6-Shogaol is the dehydrated form of another ginger compound called 6-gingerol. It contains a phenolic ring that contributes to its antioxidant and anti-inflammatory properties, a ketone group that gives it its pungent flavor, and an alkyl chain that makes it soluble in fat, facilitating its absorption into the body [21].

Several investigations have shown that 6-shogaol has a strong anti-inflammatory effect [26–29]. According to some reports, ginger’s 6-shogaol is its most effective anti-inflammatory and antioxidant substance [29, 30]. Moreover, ginger extract and its bioactive components, 6-shogaol, and gingerols, show a variety of pharmacological properties, including anti-emesis, [31] anti-tumor [32], and analgesic effects [29, 33] in numerous diseases. Prevailing research has demonstrated that 6-shogaol treatment prevents in-vitro neuronal cell death and improves rat motor neuronal repair [34, 35]. 6-shogaol helps reduce inflammation by suppressing TNF-α, IL-8, and IL-6. In addition, 6-shogaol was discovered to block the nuclear factor kappa B (NF-kB) p65 triggered by preventing IkB-α from being phosphorylated and degraded, which in turn prevents the generation of pro-inflammatory indices with the help of JNK regulation [26]. In this research, the evidence points towards the influence of 6-shogaol against cognitive disorders but, no evidence indicates the influence of 6-shogaol against the 3-NPA-induced HD rat model. Therefore, an attempt has been made to assess the protective effect of 6-shogaol against 3-NPA-induced HD and this is the novelty of this research.

## Materials and methods

### Animals

A total of thirty Male Wistar rats weighing between 175±25 g were used. The rats were obtained from T. G. Lab, M.S., India, and housed in a standard working room setting with food and unrestricted water use during the natural hours of the day (dark and light cycle). The necessary approvals were obtained in accordance with the Institutional Animal Ethics Committee, India (IAEC/TRS/PT/22/27) and the research was conducted as per the ARRIVE guidelines [36]. Prior to the investigation, all animal groups underwent a controlled seven-day acclimatization phase in the laboratory.

### Drugs

The following materials, all of the analytical grade and from trustworthy sources, were used in this work: purchase of 3-NPA from MSW Pharma, India; 6-shogaol (Yucca enterprises, India); and kits for IL-β (IL-1β, KLR0119), IL-6 (KB3068), TNF-α (KB3145), BDNF (KLR2095), and nuclear factor erythroid 2-related factor (Nrf2, KLR1083) which were examined by rat enzyme-linked immune-sorbent examine kit [ELISA, Krishgen, India].

### Experimental design

**Fig 1:**
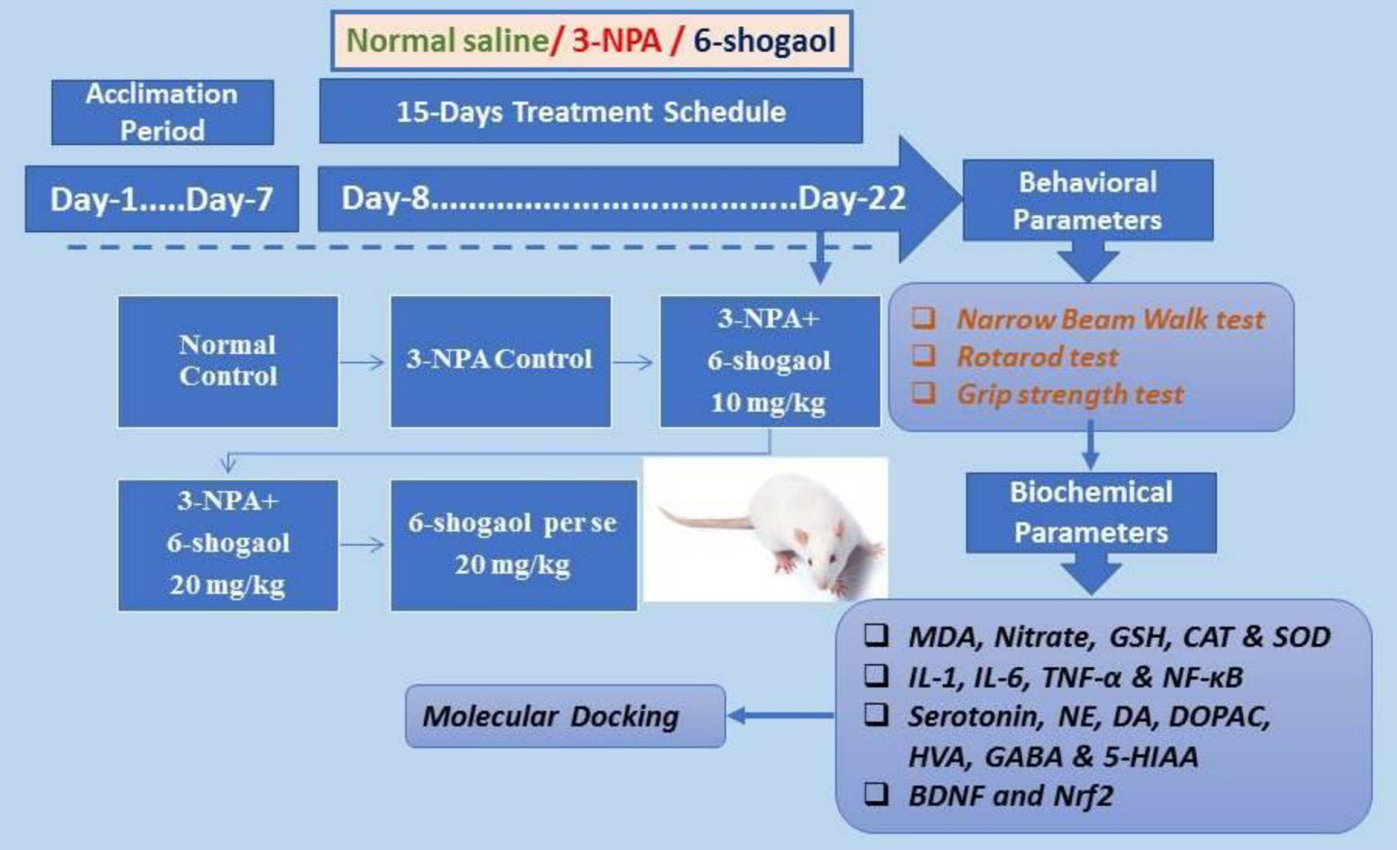
Experimental design.

The research is based on earlier completed research, which has been slightly modified [37, 38]. In the experiment, rats were assigned to five random groups. (Fig 1)

Group 1: received normal saline (control) at a dose 3 mL/kg orally,

Group 2: received 3-NPA at a dose 10 mg/kg i.p.

Group 3: received 3-NPA + 6-shogaol at a dose of 10 mg/kg orally,

Group 4: received 3-NPA + 6-shogaol at a dose of 20 mg/kg orally,

Group 5: received 6-shogaol at a dose of 20 mg/kg orally.

After one hour of treatment with 3-NPA at a dose of 10 mg/kg, intraperitoneally, the normal treated group received 3 ml/kg normal saline, while the 3-NPA control group received 3 ml/kg p.o. 0.5% sodium carboxy methyl cellulose (CMC) daily from Day 8 to 22. 6-Shogaol (prepared in normal saline solution) was administered orally in the morning to the test group at a dose of 10 or 20 mg/kg for 15 days [39, 40]. Group-3 and 4 received 3-NPA at a dose of 10 mg/kg (i.p.) daily, an hour after the above oral treatment with 6-shogaol. The rats were used for the behavioral tests. All the behavioral tests were performed in the morning session. After completion of the behavioral test, animals were subjected to anesthesia using an intraperitoneal injection of ketamine 75 mg/kg (Ciron Drugs & Pharmaceuticals Pvt Ltd., M.S., India) and xylazine 10 mg/kg (PCI, Bengaluru, Karnataka, India). Subsequently, euthanasia was performed via cervical dislocation. The brain tissue was used for biological evaluations at the end of the study [41].

### Behavior paradigm

#### Narrow beam walk

In the behavioral study, a narrow beam walk test was used to assess the rats’ balance and quick motor coordination. The apparatus’ basic design consisted of a wooden beam with measurements of 150 × 4 × 3 that was mounted 80 cm above the floor and supported by two wooden pillars that held both ends of the beam in place. The rats were exercised to walk across this wooden beam from one end to the other for two minutes. The procedure involved considering the total time taken to cross the designated beam while making the assessment [38].

### Rotarod

The goal of this test is to evaluate the animals’ balance and motor coordination. Each animal was given five experimental days before the start of the experiment. In the current study, animals that could stay on a rod, with a diameter of 3 cm and with a height of 120 cm that rotated at 20 rpm for five minutes were used. Following the open field study, the fall off latency was noticed on test day [42].

### Grip strength test

It was thought that the latency of grasping a horizontal wire served as a surrogate for grip strength. Each animal was allowed to dangle from a 2 mm wide by 35 cm long steel wire that was stretched horizontally over a foam support at a height of 50 cm using its forepaws. Each animal was timed while grasping the wire and the duration was observed.

### Biochemical test

#### Brain homogenate

The brain tissue of the animals was dissected and washed with ice-cold isotonic saline. To homogenize the isolated brain samples, 0.1 M a phosphate buffer with pH 7.3 was used. The tissues were centrifuged, and the supernatant was used for the biochemical analysis [41, 43].

### Biochemical assessment

In the current study, various brain biochemical tests were performed to determine the role that different biomarkers play in the etiology of HD.

### GSH

The amount of GSH in the brain is measured by precipitating one milliliter of trichloroacetic acid with an equivalent volume of brain homogenate. Phosphate buffer solution (PBS) and 5-nitro-bis (2-nitro-benzoic acid), popularly known as the DTNB reagent, were added to the supernatant to enrich it. The absorbance at 412 nm was measured with a spectrophotometer. A standard curve was used to determine the quantity of GSH that was present. The results were expressed in terms of GSH concentration micromoles per milligram (μmoles/mg) [44].

### CAT

In the tests, 50 nM concentrations of phosphate buffer and homogenate of brain supernatant are utilized. After adding H2O2, spectrophotometry was used to measure the mixture’s absorbance at intervals of 15 seconds at a wavelength of 240 nm. The activity was measured and quantified in units of micromoles per milligram (μmoles/mg) [45].

### Malonaldehyde (MDA)

The separated supernatant is subjected to treatment with TBARS solution and trichloroacetic acid in order to carry out the investigation. The solution is thereafter heated for a duration of 90 minutes and expeditiously cooled using cold water. The solution underwent centrifugation with a force of 1500 ¼ g for a minimum duration of 15 minutes. Subsequently, the resultant mixture was subjected to spectrophotometric analysis at a wavelength of 532 nm. The quantification of MDA concentration in brain tissue was conducted by measuring the amount of MDA in nanomoles per milligram (nmoles/mg) [46].

### Nitrate

Nitrite, a nitric oxide (NO) production marker, was quantified using the colorimetric method. Nitrite concentrations were calculated using nitrite calibration curve reported in μg/mL [47].

### SOD

The liquid portion was subjected to a 30-minute treatment in a potassium phosphate buffer by the utilization of xanthine oxidase and xanthine. The mixture was forcefully combined with Nitro-blue tetrazolium (NBT) to produce a blue formazan combination. The material’s wavelength was determined to be precisely 550 nm through the application of spectrophotometry. The amount of protein needed to avoid a 50% decrease in NBT is equivalent to one unit of SOD activity (U/mg) [48].

### Neuroinflammatory marker

A kit for immunoassay was utilized to measure the activity of the pro-inflammatory markers IL-1β, NF-κB, IL-6, and TNF-α. Indicator concentrations were calculated from calibration curves and expressed as ng/mL protein.

### Estimation of BDNF and Nrf2 activity

To assess BDNF and Nrf2 in the homogenate of brain tissue, the manufacturer recommends the use of rat ELISA kits.

### Assessment of monoamines

Various neurotransmitters including GABA, dopamine (DA), 5-hydroxyindoleacetic acid (5-HIAA), norepinephrine (NE), 3,4-dihydroxyphenylacetic acid (3-DOPAC), serotonin (5-HT), and homovanillic acid (HVA) have been measured using high-performance liquid chromatography (HPLC Agilent 1100, Agilent Technologies Helwette-Packard-Strasse 876337, Waldbronn, Germany) [41, 49].

### Molecular docking

AutoDockTools, Chimera, and Maestro were used to detect the receptor grid. The prepared proteins (2AZ5, 1SVC, 1ALU and 6Y8M) were displayed in the workspace. For the protein having co-crystal ligands, volume of grid was identified using dimensions of cocrystal ligand. Meanwhile, those without cocrystal ligands were calculated using Computed Atlas of Surface Topography of proteins (CASTp) server.

Ligand molecules were designed in MarvinSketch v21.13 (targeted molecule imported), cleaned in 2D and 3D formats, and minimized using MMFF94 force field. The lowest energy conformer was selected, which was then saved in 3D mol2 file format.

After obtaining the ligand and protein, their structures were converted to pdbqt format using in-house bash script made via AutoDock tools 1.5.7 for ligand and ADFRsuit for proteins. All the rotatable bonds of ligands were allowed to rotate freely, and the receptor was considered rigid. For docking studies, the researchers used the AutoDock Vina 1.2.5, with 0.375 ˚A of spacing between the grid points. The grid box was centered on the active site of the target, allowing the program to search for additional places of probable interactions between the ligands and the receptor. Other configurations were considered default. The XYZ coordinates and size of the grid box in Table 3. Other parameters like CPU were set to 23, exhaustiveness was 32, number of modes was 9, and energy range was set to 3. Redockings were performed with the same configurations of the previously performed dockings. Results obtained were subjected to make a complex 2D and 3D images using Biovia Discovery Studio visualizer.

### Statistical

The results are given as mean ± SEM. The researchers performed the normality pass test by using Shapiro-Wilk Test. One-way analyses of variance followed by the Tukey’s post hoc test were performed for numerical expressive style (GraphPad Prism 8.0.2).

## Results

### Behavioral test

#### Narrow beam walk

**Fig 2:**
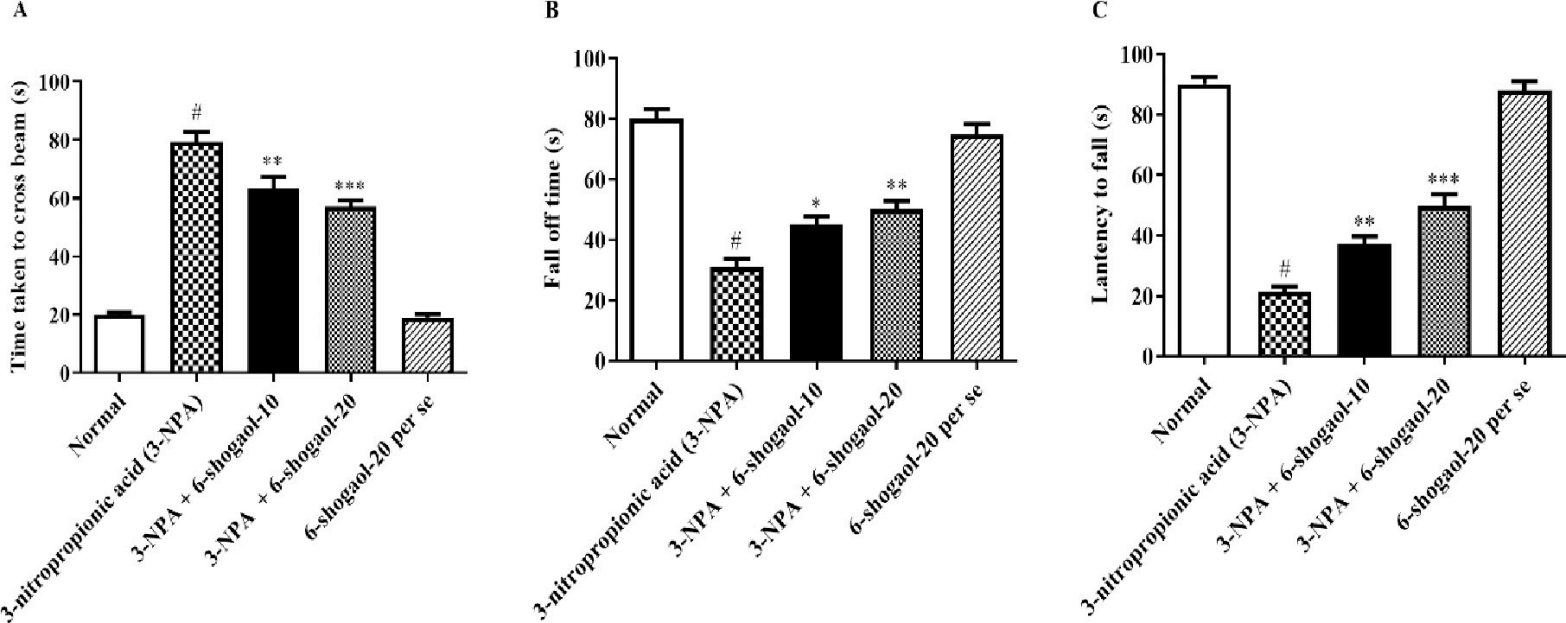
(A-C). Effect of 6-shogaol on A) Narrow Beam Walk, B) Rotarod, and C) Grip strength test. Mean ± S.E.M. (n = 6). #P < 0.001 vs. Normal control, *P< 0.05, **P<0.01 and ***P<0.001 vs 3-NPA control. One-way ANOVA was followed by Tukey’s test.

The impact of 6-shogaol on the rats’ behavior during the narrow beam walk is shown in Fig 2A. 3-NPA control rats appropriated substantially more time to reach the wooden platform in comparison to the normal control group, indicating that the 3-NPA induced symptoms similar to HD. The time needed to walk transversely the beam was considerably reduced in the 6-shogaol-administered group in comparison to the 3-NPA control [F (4, 25) = 103.1, P<0.0001], demonstrating the restoration of their normal cognitive performance. Treatment with 6-Shogaol per se group had no significant influence on the narrow beam walk test compared to normal control group.

### Rotarod and grip strength test

Severe muscular weakness and locomotor impairment were caused by 3-NPA injection. 3-NP-injected rats markedly reduced the fall-off latency and the amount of time required to hold the wire during the grip strength and rotarod tests, respectively, when associated to normal control group. Treatment with 6-shogaol reduced these motor deficits, as seen in the significant increase in the 3-NPA groups, the time spent gripping the wire [F (4, 25) = 121.4, P<0.0001], and rotarod fall-off latency [F (4, 25) = 48.28, P<0.0001]. 6-Shogaol per se group had no significant influence on motor deficits and grip strength compared to the normal control group. (Fig 2B and 2C).

### Biochemical test

#### MDA, nitrate, SOD, GSH, CAT

**Fig 3:**
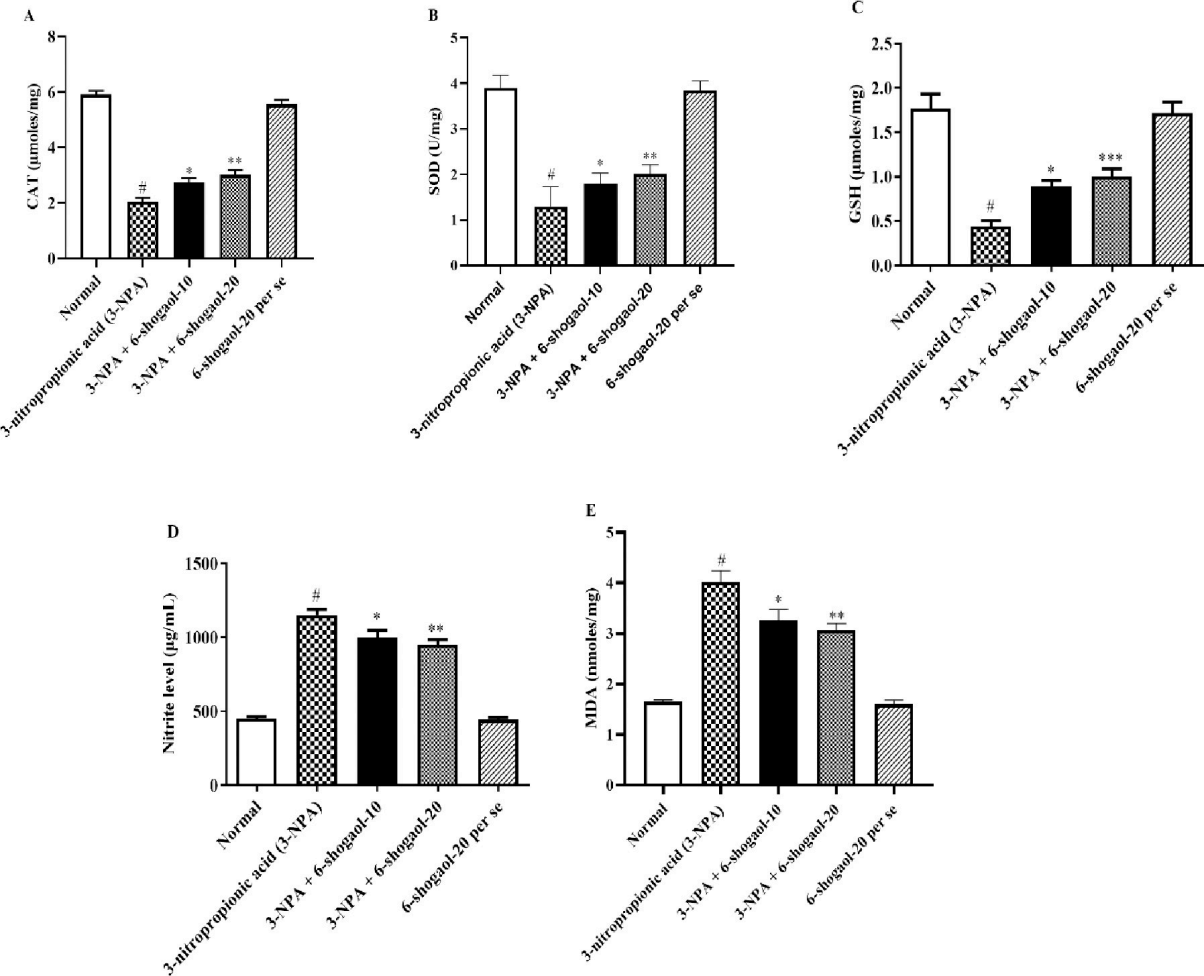
(A-E). Effect of 6-shogaol on A) CAT, B) SOD, C) GSH, D) Nitrate, and E) MDA level. Mean ± S.E.M. (n = 6). #P < 0.001 vs. Normal control, *P< 0.05, **P<0.01 and ***P<0.001 vs 3-NPA control. One-way ANOVA was followed by Tukey’s test.

Rats administered with 3-NPA showed significantly enhanced nitrate and MDA contents with significantly lower CAT, SOD, and GSH content compared to rats in normal control group. 6-shogaol minimized the oxidative stress caused by 3-NPA with substantial rise in levels of CAT [F (4, 25) = 132.9, P<0.0001], SOD [F (4, 25) = 111.4, P<0.0001], and GSH [F (4, 25) = 28.38, P<0.0001], although contents of nitrate [F (4, 25) = 100.4, P<0.0001] and MDA [F (4, 25) = 46.15, P<0.0001] were decreased concerning the 3-NPA group, respectively. Treatment with the 6-Shogaol per se group had no significant influence on CAT, SOD, GSH, MDA, and Nitrate test compared to the normal control group (Fig 3A–3E).

### DA, 5-HT, NE, and GABA

**Fig 4:**
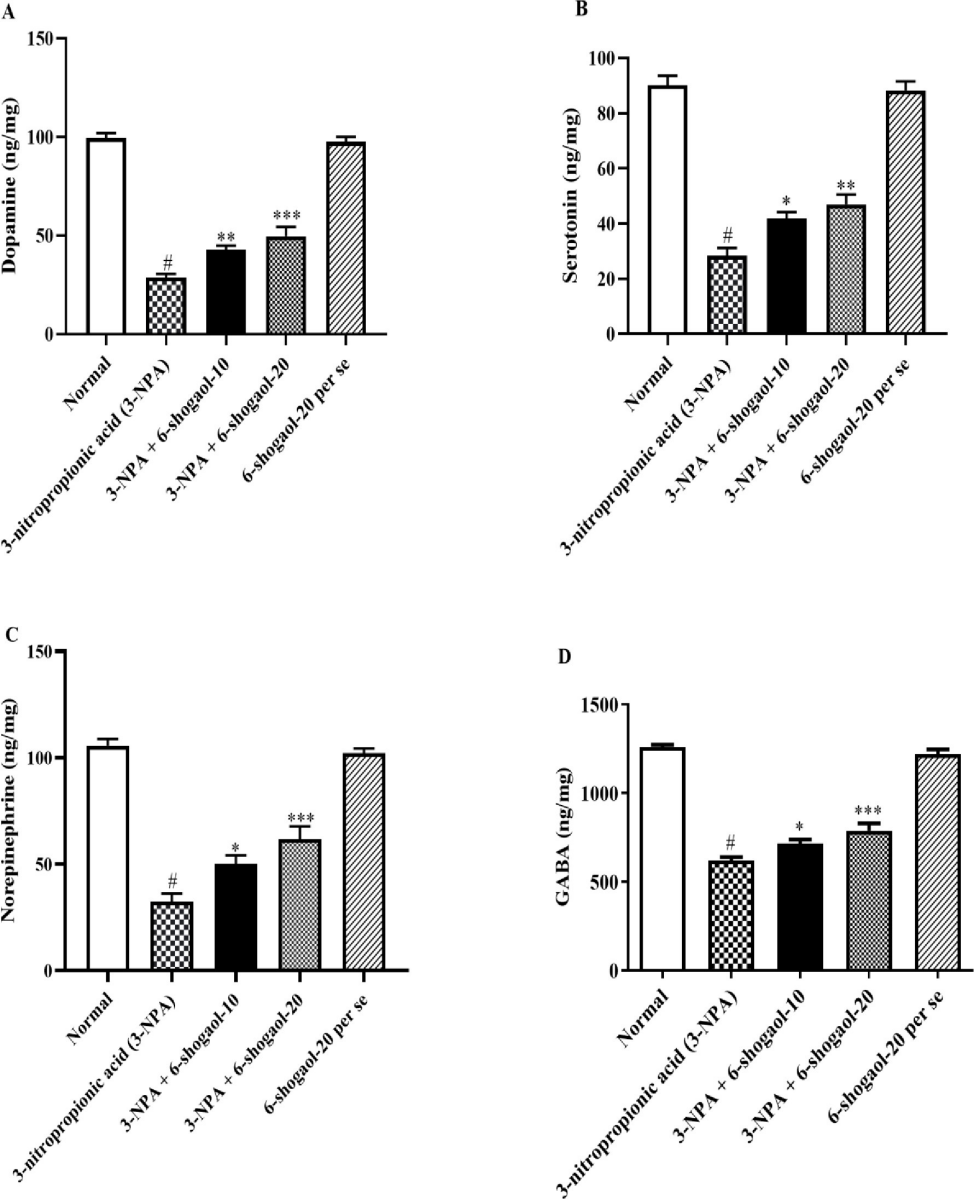
(A-D). Effect of 6-shogaol on A) DA, B) 5-HT, C) NE, and D) GABA content. Mean ± S.E.M. (n = 6). #P < 0.001 vs. Normal control, *P< 0.05, **P<0.01 and ***P<0.001 vs 3-NPA control. One-way ANOVA was followed by Tukey’s test.

Rats administered with 3-NPA showed dramatically reduced contents of DA, 5-HT, NE, and GABA, respectively, in comparison to the normal control group. 6-shogaol markedly enhanced the contents of DA [F (4, 25) = 129.4, P<0.0001], 5-HT [F (4, 25) = 84.26, P<0.0001], NE [F (4, 25) = 64.08, P<0.0001], and GABA [F (4, 25) = 128.5, P<0.0001] when compared to the 3-NPA group. This mitigated the neurotransmitter levels that 3-NPA had generated. Treatment with the 6-Shogaol per se group had no significant influence on neurotransmitter levels compared to the normal control group (Fig 4A–4D).

### HVA, DOPAC, and 5-HIAA levels

**Fig 5:**
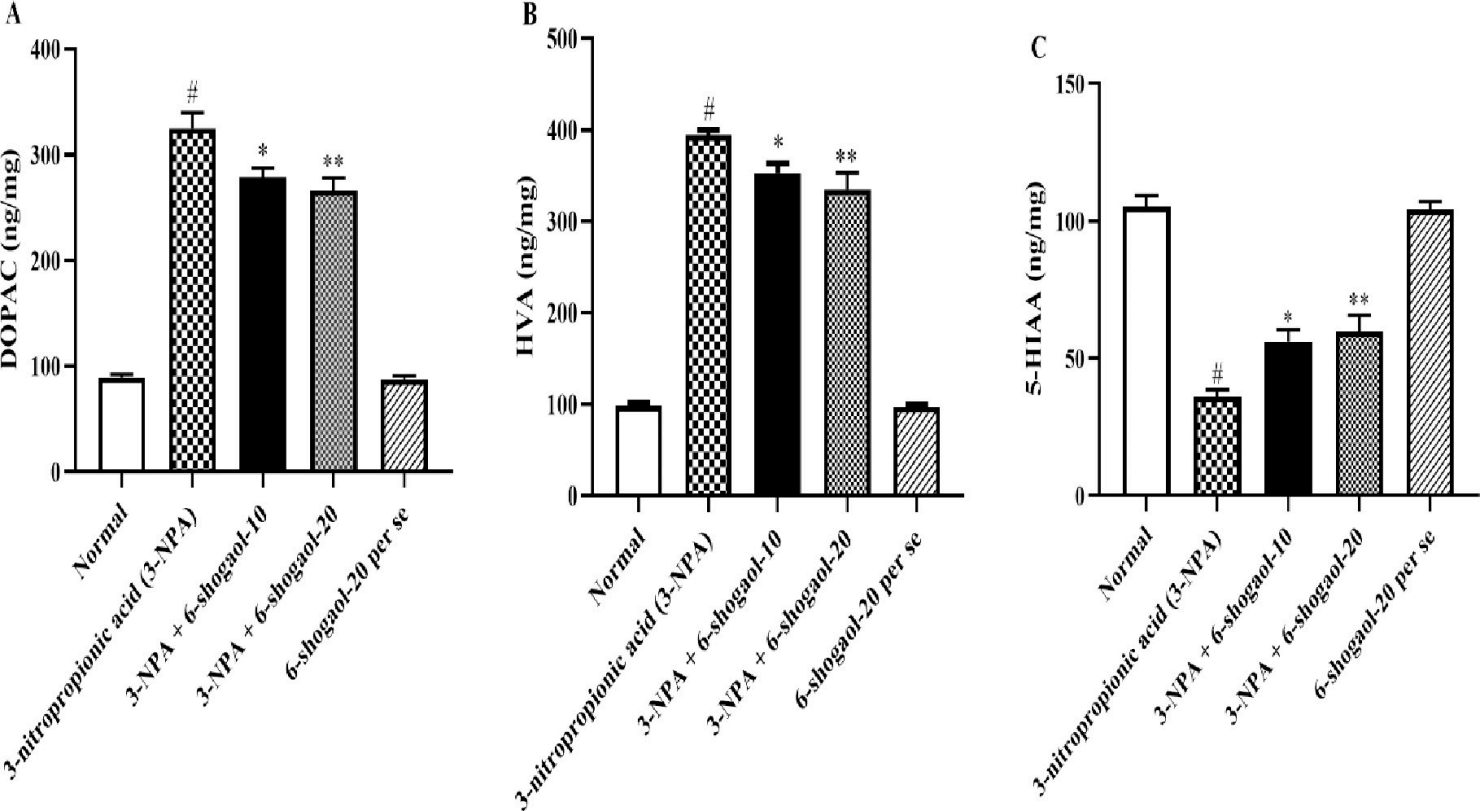
(A-C). Effect of 6-shogaol on A) DOPAC, B) HVA, and C) 5-HIAA content. Mean ± S.E.M. (n = 6). #P < 0.001 vs. Normal control, *P< 0.05 and **P<0.01 vs 3-NPA control. One-way ANOVA was followed by Tukey’s test.

Rats administered with 3-NPA had noticeably higher contents of DOPAC and HVA and significantly lower levels of 5-HIAA in comparison to rats in the normal control group. 6-shogaol markedly reduced DOPAC [F (4, 25) = 142.7, P<0.0001] and HVA [F (4, 25) = 209.0, P<0.0001] levels, although 5-HIAA [F (4, 25) = 54.40, P<0.0001] contents considerably increased than those of the 3-NPA group. 6-Shogaol per se group had no significant influence on HVA, DOPAC, and 5-HIAA levels compared to the normal control group (Fig 5A–5C).

### IL-6, IL-1β, and TNF-α

**Fig 6:**
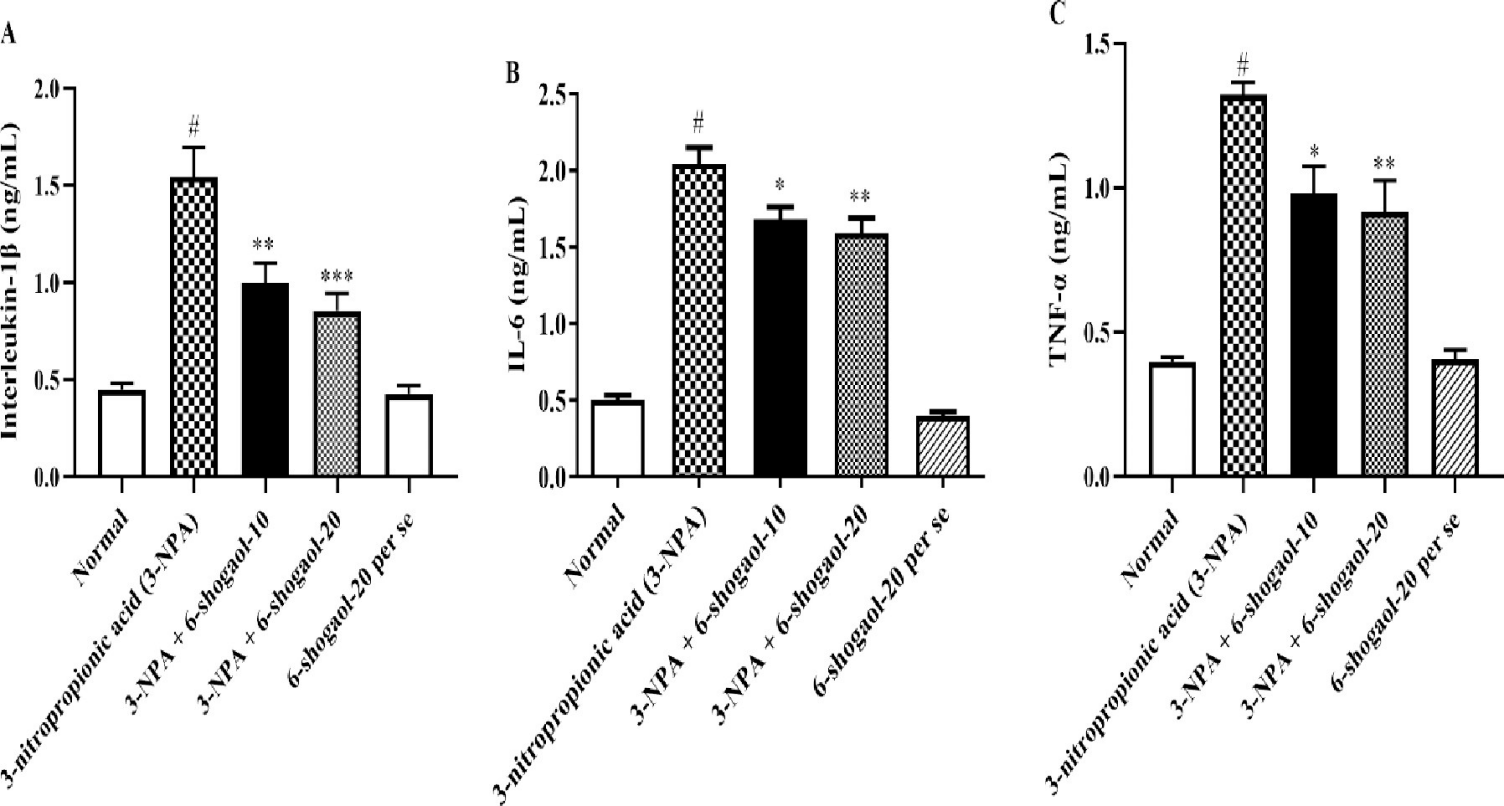
(A-C). Effect of 6-shogaol on A) IL-1β, B) IL-6, and C) TNF-α level. Mean ± S.E.M. (n = 6). #P < 0.001 vs. Normal control, *P< 0.05, **P<0.01 and ***P<0.001 vs 3-NPA control. One-way ANOVA was followed by Tukey’s test.

Rats administered with 3-NPA showed significantly more IL-6, IL-1β, and TNF-α compared to the normal control group. However, 6-shogaol significantly decreased the amounts IL-1β [F (4, 25) = 23.49, P<0.0001], IL-6 [F (4, 25) = 98.52, P<0.0001], and TNF-α [F (4, 25) = 33.13, P<0.0001] associated to 3-NPA group. Whereas 6-Shogaol per se group had no significant influence on IL-6, IL-1β, and TNF-α levels compared to the normal control group (Fig 6A–6C).

### NF-κB, BDNF, and Nrf2 contents

**Fig 7:**
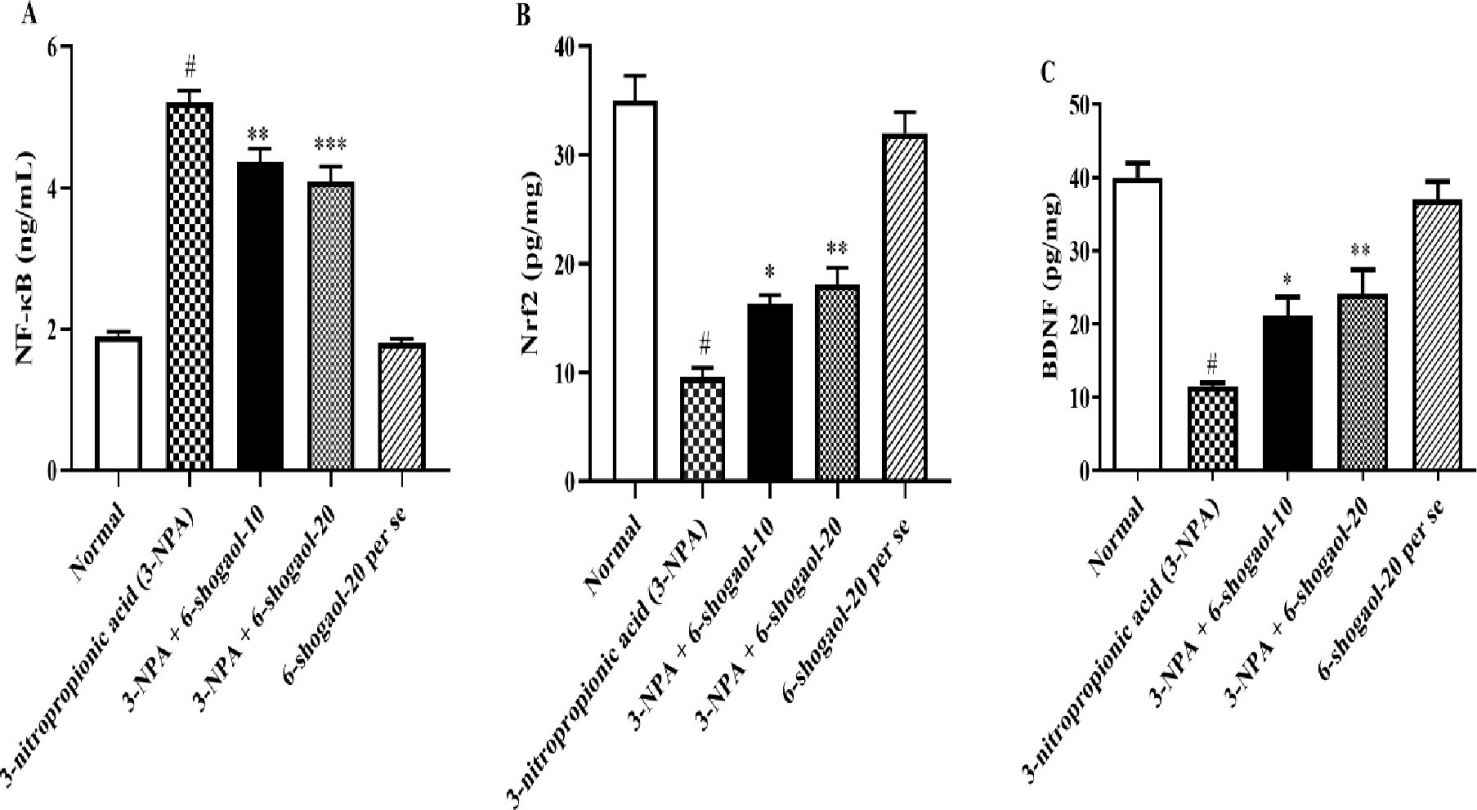
(A-C). Effect of 6-shogaol on A) NF-κB, B) Nrf2, and C) BDNF level. Mean ± S.E.M. (n = 6). #P < 0.001 vs. Normal control, *P< 0.05, **P<0.01 and ***P<0.001 vs 3-NPA control. One-way ANOVA was followed by Tukey’s test.

Rats administered with 3-NPA had significantly higher NF-κB levels, whereas levels of Nrf2 and BDNF were significantly lower than the normal control group of rats. 6-shogaol significantly decreased NF-κB [F (4, 25) = 100.6, P<0.0001] level, while Nrf2 [F (4, 25) = 46.66, P<0.0001] and BDNF [F (4, 25) = 25.67, P<0.0001] levels were significantly reduced, relative to the 3-NPA group, respectively. Treatment with the 6-Shogaol per se group had no significant influence on NF-κB, BDNF, and Nrf2 Contents compared to the normal control group (Fig 7A–7C).

### Molecular docking

**Fig 8:**
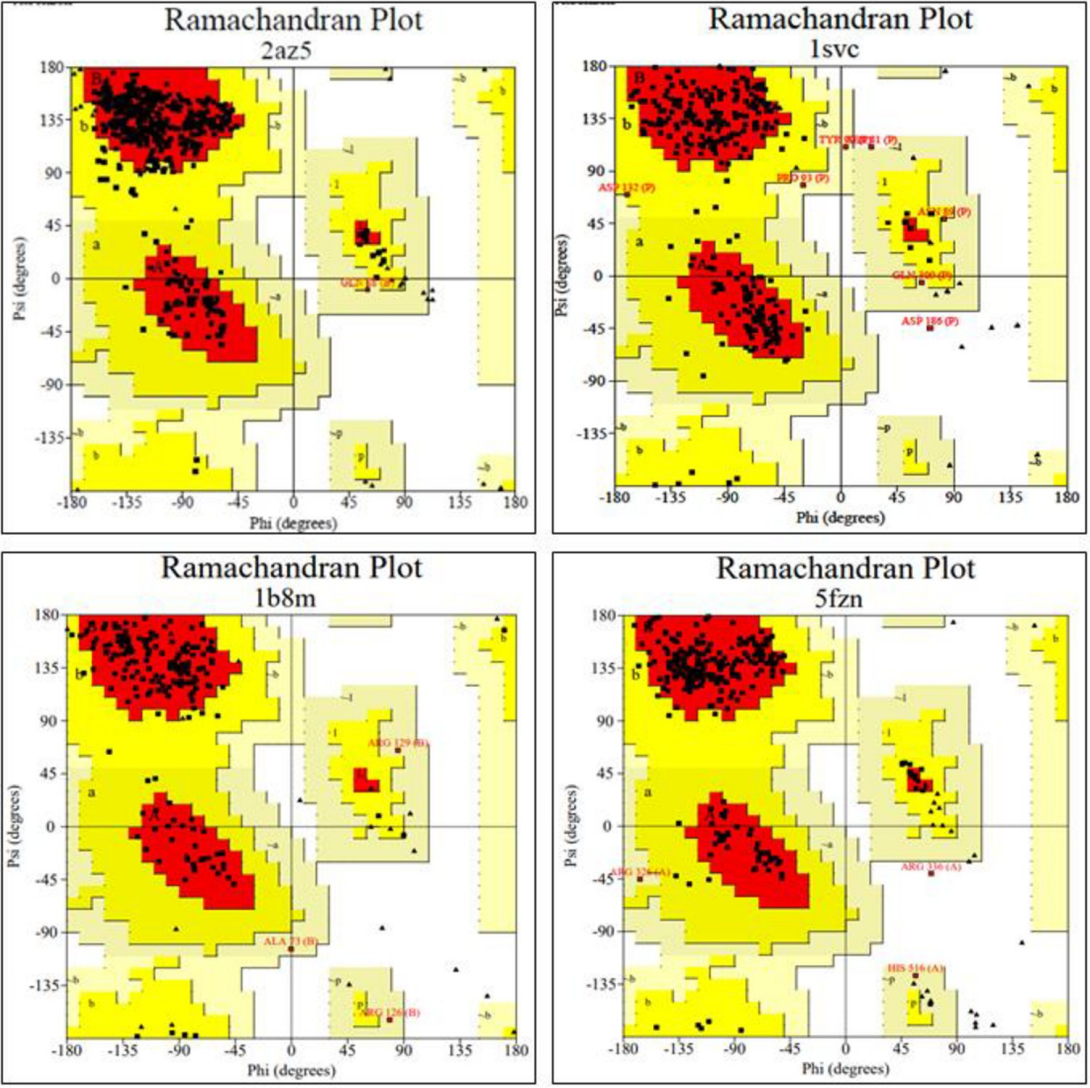
Ramachandran Plot 2AZ5, 1SVC, 1B8M, 5FZN obtained from PROCHECK server.

Molecular docking study was performed to estimate the preventive effect of 6-shogaol on HD induced by 3-NPA by moderating TNF-*α*, NF-κB, BDNF, and NRF2 signaling pathways. 6-shogaol showed major target to TNF-*α*, NF-κB, BDNF, and NRF2 in 3-NPA-instigated HD. Therefore, the fast sequence of TNF-α, NF-Kβ, BDNF, and Nrf2 for homo sapience taxon was retrieved from National Centre for Biotechnology Information (NCBI) server and similar biological sequences available in the Protein Data Bank (PDB) were searched using Basic Local Alignment Search tool (BLAST), where the researchers sorted top five to ten selected sequences in terms of best query coverage, percentage identity, and E- value. Three-dimensional structures of TNF-α, NF-κB, BDNF, and Nrf2 were retrieved from the PDB and rigorously validated. Mutation, wwPDB Validation, co-crystal ligand and Ramachandran plot. Details are represented in Table 1 and Fig 8.

**Table 1:** Standard values and protein retrieved for validation of docking study selection.

Parameters	Protein Details				Standards
Targets	TNF-α	NF-κβ	BDNF	Nrf2	-
Protein ID	**2AZ5**	**1SVC**	**1B8M**	**5FZN**	-
Method of Experiment	X-RAY Diffraction	X-RAY Diffraction	X-RAY Diffraction	X-RAY Diffraction	X-RAY Diffraction
Mutation	No	No	No	No	No
Resolution	2.10 Å	2.60Å	2.75Å	1.97Å	Near about 2.00 A^0^
wwPDB Validation	Better	Better	Better	Better	Better
Co-Crystal Ligand	307	Absent	Absent	FB2	-
Ramchandran Plot (by PROCHECK server) Residues in favoured + Allowed regions	90.2%	85%	89.5%	90.0%	>80%

A workspace with the prepared proteins (2AZ5, 1SVC, 1ALU and 6Y8M) was displayed. Using cocrystal ligand dimensions, volume of grid was identified for proteins with cocrystal ligands, whereas for proteins without cocrystal ligands, CASTp server (Computed Atlas of Surface Topography of Proteins) was used. The amino acids identified in grid pockets represented in Table 2

**Table 2:** Active sites amino acids.

Protein	Active Sites Amino Acids
**2AZ5**	LEU57A, TYR59A, SER60A, TYR119A, LEU120A, GLY121A, GLY122A, TYR151A, LEU57B, TYR59B, SER60B, TYR119B, LEU120B, GLY121B, TYR151B, LEU55D
**1SVC**	LYS52, LYS52, ARG54, ARG54, ARG54, ARG54, ARG54, GLY55, GLY55, SER243, ALA248, SER249, ASN250, ASN250, ASN250, LEU251, LEU251, LEU251, LEU251, ARG336, GLU341, GLU341,THR342, SER343, GLU344, GLU344, GLU344.
**1B8M**	VAL42, PRO43, VAL44, SER45, LYS46, LEU49, LYS50, GLN51, TYR52, PHE53, TYR54, SER85, TYR86, VAL87, ARG88, ALA89, LEU90, ILE98, GLY99, TRP100, PHE102, ILE105, THR107, PRO45, ALA46, ALA47, LEU52, ARG53, GLN54, TYR55, PHE56, PHE57, SER95, TYR96, VAL97, ARG98, ALA99, LEU100, VAL108, GLY109, TRP110, TRP112, ILE115, THR117
**5FZN**	TYR334A, TYR334A, SER363A, SER363A, GLY364A, GLY364A, ARG415A, ARG415A, ARG415A, ARG415A, ARG415A, ALA556A, ALA556A, SER602A, SER602A, SER602A, GLY603A, GLY603A, GLY603A

A small enclosing box was designed to match the active site’s shape and character, as well as the docking ligands expected to be present Table 3.

**Table 3:** Grid parameter.

PDB ID	CENTRE CO-ORDINATES			SIZE CO-ORDINATES		
	X	Y	Z	X	Y	Z
**2AZ5**	-19.41	-74.65	33.85	20	20	20
**1SVC**	34.065	11.277	37.594	30	30	30
**1B8M**	-1.906	33.677	4.202	20	20	20
**5FZN**	14.02	66.12	30.64	20	20	20

In the NF-κβ (1SVC), the interaction was through hydrogen and hydrophobic bonds in 6-shogaol. The 6-shogaol showed the favorable binding energies at -6.866 (NF-Kβ) kcal/mol.

The intermolecular interactions 6-shogaol on ligands TNF-α (2AZ5) and Nrf-2 (5FZN) showed the binding energies at -6.657 (TNF-α) and -6.626 (NrF-2) kcal/mol (Table 4).

**Table 4:** Docking score and intermolecular interactions of ligands TNF-α (2AZ5), NF-Kβ (1SVC), BDNF(1B8M), and NRF-2(5FZN).

Sr. No	Molecule	Binding Energy (Kcal/Mol)	Type Of Interaction	Residue Id	Distance (Å)
1	1SVC-6-SHOGAOL	-6.866	Hydrophobic Interactions	LYS52P	3.91
				PRO71P	3.9
				LYS80P	3.8
				TYR82P	3.68
			Hydrogen Bonds	SER81P	2.13
				SER81P	1.87
2	2AZ5-6-SHOGAOL	-6.657	Hydrophobic Interactions	LEU57A	3.59
				LEU57B	3.55
				LEU57B	3.73
				TYR59B	3.73
				TYR119A	3.56
				TYR119B	3.86
			Hydrogen Bonds	TYR151A	2.74
				TYR151A	2.21
			π-Stacking	TYR119A	4.24
3	1B8M-6-SHOGAOL	-9.271	Hydrophobic Interactions	GLN51A	3.65
				GLN54B	3.64
				PHE56B	3.93
				PHE56B	3.83
				ARG88A	3.75
				VAL97B	3.9
				VAL97B	3.88
				ARG98B	3.58
				TRP100A	3.4
				TRP100A	3.56
				PHE102A	3.82
				ILE115B	3.76
			Hydrogen Bonds	GLN51A	2.94
				TYR52A	2.55
4	5FZN-6-SHOGAOL	-6.626	Hydrophobic Interactions	TYR334A	3.69
				TYR334A	3.86
				TYR334A	3.71
				ARG415A	3.58
			Hydrogen Bonds	VAL463A	2.22

**Fig 9:**
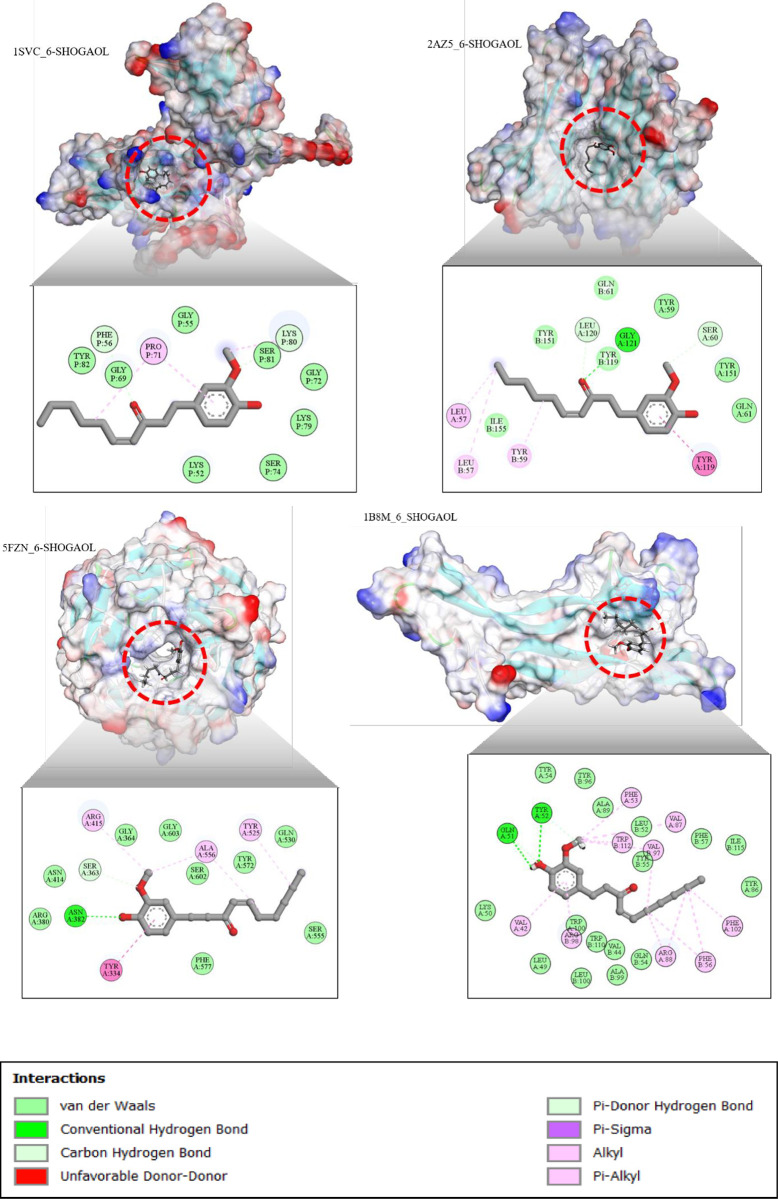
Molecular docking of the 6-shogaol with TNF-α (2AZ5), NF-Kβ(1SVC), BDNF (1B8M), and NRF-2(5FZN) proteins.

The 6-shogaol showed favorable docking energy to BDNF (1B8M). Different hydrogen and hydrophobic bonds were observed as a result of the interaction of 6-shogaol and BDNF (1B8M), as shown in Fig 9. The 6-shogaol showed favorable negative binding energies at -9.271 (BDNF) kcal/mol

## Discussion

The current work highlighted the neuroprotective properties of 6-shogaol in rodent models of 3-NPA-induced HD by targeting pro-inflammatory cytokines and the NF-κB NF-κB-BDNF-Nrf2 pathway. As previously mentioned, 3-NPA injection causes striatal neurodegeneration, which significantly impairs an animal’s ability to hold objects, move around, and coordinate movements [42]. Accordingly, the rotarod paradigm and the grip strength paradigm conducted in the current study revealed decreased grip strength, disrupted locomotor function, and decreased motor coordination in the 3-NPA-induced HD rats. Interestingly, 6-shogaol treatment improved the locomotor behavior, grip strength, and muscle performance of 3-NPA-induced HD rats in the relevant tests indicated above as well as their capacity to maintain their position for an enhanced period of time with the rotarod test.

A status of oxidative stress was evident in the current study’s 3-NPA-induced HD rats, as shown by a notable reduction in SOD, GSH, and CAT contents, in contrast to an increase in nitrate and MDA levels. The 6-shogaol therapy dramatically improved the levels of nitrite and the indicators for oxidative stress. Previously published studies have highlighted the involvement of a number of biochemical factors that exacerbate the development of HD [50–52]. The biochemical investigations essentially involved the evaluation of oxidative stress parameters, quantification of numerous brain enzymatic reactions, proinflammatory mediators estimation, and analysis of the production of several amines. Moreover, prior research revealed considerable changes in SOD, MDA, CAT, GSH, and nitrite content in the brain and neural tissues; these changes were linked to HD [37, 50, 51, 53].

According to previous findings, neurotransmitter levels changed as an obvious occurrence after chemically induced neurotoxicity. Moreover, monoamines glutamate, GABA, 5-HT, DA, and NE were recognized as key targets in the pathogenesis of HD [6, 7, 54, 55]. It has been repeatedly demonstrated that the human HD brain suffers from dendritic spine loss and substantial GABA-ergic cell impairment [56, 57]. Damage ranged from a simple loss of dendritic spines to complete loss of striatal medium spiny neurons in the 3-NPA-induced HD rat model [51, 58, 59]. It has been noted that after constant systemic infusion or after intra-striatal infusion of 3-NPA in rats over the course of five days, striatal GABA was dramatically reduced, but not glutamate [58]. Research was undertaken to evaluate how many free amino acids and monoamines were present in brain tissue homogenate. Neurotransmitters such as NE, GABA, DOPAC, 5-HT, HVA, 5-HIAA, and DA were significantly altered by 3-NPA-induced HD rats. However, 6-shogaol therapy dramatically improved the aforementioned parameters in rats.

ROS may be produced as a result of the mitochondrial deficiency, which enhances oxidative stress [53, 60, 61]. and reduces antioxidants, leading to an increase in proinflammatory cytokines and brain injury and a decline in HD [62]. In response to neurodegeneration and neurotoxicity, cytokines (IL-1β and TNF-α) were activated, raising the risk of illness [63]. Injury to GABA-ergic MSN caused by 3-NPA [64] elevated pro-inflammatory cytokines as a result. The researchers discovered that 6-shogaol doses markedly decreased the number of cytokines (IL-6, NF-κB, IL-1β and TNF-α) in 3-NPA-induced HD rats.

One possible explanation for the enhanced oxidative status and neuronal degeneration caused by 3-NPA injection is the clear inhibition of the BDNF/Nrf2 antioxidant mechanism shown in this study [65]. Since Nrf2 is known to control the synthesis of GSH and enzymes that are connected to it, such as glutathione S-transferases (GST), it may be able to counteract the increased oxidative stress caused by 3-NPA [66]. Moreover, Nrf2 blocks NOX function, preventing ROS synthesis by this enzyme, which is important for ROS formation [67]. BDNF, a crucial neurotrophic protein involved in memory and learning processes as well as neuronal growth, differentiation and survival has also been demonstrated to be transcriptionally induced by Nrf2 [68, 69]. Meanwhile, Nrf2 translocation and subsequent activation, which result in corrected redox clot formation are mediated by BDNF [70]. In HD animal models, BDNF protein levels are decreased, according to a prior publication. [71, 72]. In the present investigation, 3-NPA-induced HD rats showed a noticeable decline in Nrf2 and BDNF levels. The treatment with 6-shogaol considerably restored the Nrf2 and BDNF levels.

Based on the docking score and intermolecular interactions of the ligands against NTNF-α (2AZ5), NF-κβ (1SVC), BDNF (1B8M), and Nrf-2 (5FZN), the ligands were found to have a high binding affinity with the target’s molecule. This shows that it can be a potential drug candidate for various diseases [73]. 6-shogaol was understood as a favorable target effect on TNF-α (2AZ5), NF-κβ (1SVC), BDNF(1B8M), and Nrf-2 (5FZN) signaling pathways that play a key role in 3-NPA-induced HD. 6-shogaol showed the best binding energies at -9.271 (1B8M_BDNF) kcal/mol. The findings from the in-silico analysis suggested that 6-shogaol could be a protective effect against 3-NPA-induced HD rats. 6-shogaol may protect against HD by inhibiting neuroinflammation (via TNF-α), activating antioxidant defenses (via Nrf-2), and enhancing neurotrophic support (via BDNF). The findings provide evidence that 6-shogaol has a protective effect against 3-NPA-induced HD in rats by targeting TNF-α, NF-κβ, BDNF, and Nrf-2 signaling pathways [74]. It is also suggested that 6-shogaol can be an effective therapeutic agent in HD treatment.

The 6-shogaol 10 and 20 mg/kg p.o. treatment group showed a significant improvement in behavioral activity over the 3-NPA-injected control rats. Moreover, 3-NPA-induced significantly altered neurotransmitters, biochemical, gene expression, and neuroinflammatory indices, which could be efficiently reversed by 6-shogaol. The 6-shogaol showed the favorable negative binding energies at -9.271 (BDNF) kcal/mol These findings also suggest that 6-shogaol may have a protective impact against 3-NPA-induced HD in the brain by improving behavioral activity, reducing oxidative stress, restoring neurotransmitters, inhibiting inflammatory markers, and downregulating NF-κB, BDNF, and Nrf2 expressions. The short duration and minimal use of animals are the study’s drawbacks. Further studies are needed to corroborate this mechanism, including the use of genetic models, western blotting, reverse transcription polymerase chain reaction and tissue immunohistochemistry. Due to the limited use of animals in this research histopathology was not performed.

## Conclusion

The current study shows that 6-shogaol decreases neurotransmitter levels and inflammatory response associated with HD in 3-NPA-injected animals, which results in behavioral and physiological changes in rats. A possible advantage of 6-shogaol is its anti-inflammatory and antioxidant properties. Future studies require the precise molecular mechanisms by which 6-shogaol exerts its neuroprotective and anti-inflammatory effects.

## Supporting information

S1 Graphical abstract (TIF)
