# Supplementary figures and images for: 6-shogaol against 3-Nitropropionic acid-induced Huntington’s disease in rodents: Based on molecular docking/targeting pro-inflammatory cytokines/NF-κB-BDNF-Nrf2 pathway
Source: PLoS One. 2024 Jul 15;19(7):e0305358. doi: 10.1371/journal.pone.0305358 (PMC11249262; doi:10.1371/journal.pone.0305358)

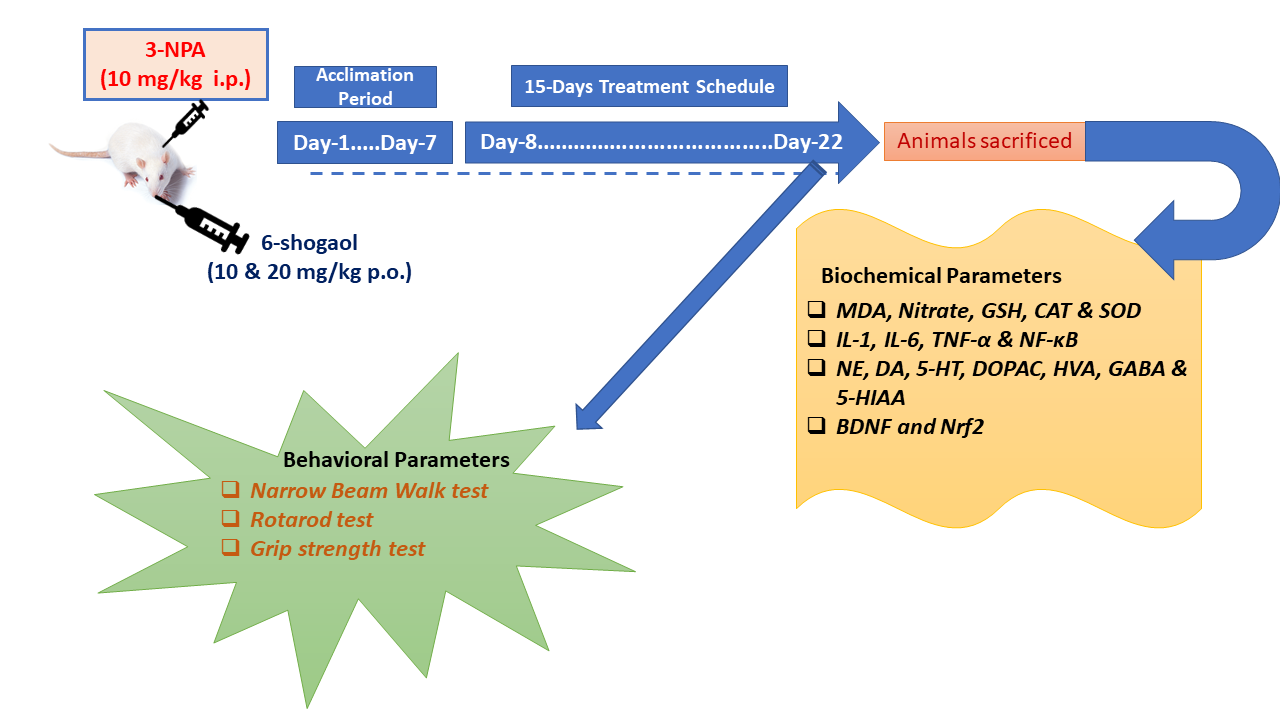

Supplement: S1 Graphical abstract — (TIF) [file pone.0305358.s001.tif]
